# Study Protocol: Efficacy of Jintiange Capsule in Lumbar Disc Herniation: A Single‐Center, Randomized Controlled Trial

**DOI:** 10.1155/prm/4091533

**Published:** 2026-10-29

**Authors:** Yinghao Zhu, Hao Lv, Yi Wang, Xunlu Yin, Kaiming Li, Hengtong He, Tianlin Wang, Long Liang

**Affiliations:** ^1^ The First Affiliated Hospital of Anhui University of Chinese Medicine Hefei China ahtcm.edu.cn; ^2^ Anhui University of Chinese Medicine Hefei China ahtcm.edu.cn; ^3^ Wangjing Hospital China Academy of Chinese Medical Sciences Beijing China cacms.ac.cn; ^4^ Xiyuan Hospital China Academy of Chinese Medical Sciences Beijing China cacms.ac.cn

**Keywords:** Jintiange capsule, low back pain, lumbar disc herniation, protocol, randomized controlled trial

## Abstract

**Background:**

Lumbar disc herniation (LDH) is a leading cause of chronic low back pain (LBP), severely impairing patients’ quality of life and imposing a substantial global socioeconomic burden. Conservative treatment remains the first‐line clinical option, but the commonly prescribed drug celecoxib carries a risk of gastrointestinal bleeding or perforation. Traditional Chinese medicine preparations have been proposed as alternatives, although current evidence is insufficient. Jintiange (JTG) capsule, developed as a substitute for tiger bone, exerts its pharmacological effects by inhibiting osteoclastogenesis and regulating the NF‐κB pathway. Previous studies have identified a close association between the p38MAPK/NF‐κB pathway and pain induced by LDH. However, the efficacy of JTG in treating LDH remains unclear. High‐quality research is thus urgently needed to provide evidence‐based support for its clinical application.

**Method:**

This randomized, single‐center clinical trial will enroll 68 participants, centrally allocated 1:1 to receive either JTG capsules plus comprehensive Western medicine (celecoxib + eperisone) or Western medicine alone. All participants will complete an 8‐week treatment phase (celecoxib for 2 weeks + eperisone for 8 weeks; with JTG capsules added for 8 weeks in the experimental group), with a follow‐up at Week 12 (4 weeks after treatment completion). The primary outcome is the change in the Japanese Orthopaedic Association (JOA) score for LBP, a validated instrument for evaluating treatment efficacy in LDH. Secondary outcomes include the Patient Global Impression of Change (PGIC), Visual Analog Scale (VAS) score, SF‐12 quality‐of‐life score, and elastic modulus of the lumbar muscles. Safety monitoring and adverse‐event recording will occur throughout the study. The primary endpoint will be analyzed using the analysis of covariance. Longitudinal VAS scores at Weeks 2, 8, and 12 will be analyzed using a mixed model for repeated measures. PGIC will be analyzed as an ordinal categorical outcome, while other secondary outcomes will be analyzed using appropriate parametric or nonparametric methods. All analyses will be performed using SPSS 28.0, with no interim analysis planned.

**Discussion:**

This trial will evaluate the clinical efficacy of JTG capsules combined with Western medicine for LBP and will explore its safety profile as a secondary/exploratory endpoint. Given the open‐label combination design, safety findings will be interpreted with caution, and causality attribution to JTG alone will be limited. The findings are expected to provide an evidence‐based treatment option, supporting the potential role of Chinese patent medicine in LBP management.

**Trial Registration:** Chinese Registry of Clinical Trials: ChiCTR2300069735

## 1. Background

Low back pain (LBP) has long been a leading cause of disability worldwide, imposing substantial social and healthcare costs. Its etiology is complex, and lumbar disc herniation (LDH) is one of the most prominent causes. Epidemiological evidence indicates that the lifetime risk for symptomatic LDH is approximately 1%–3%, with an annual incidence of 5–20 cases per 1000 adults [1, 2]. LDH is most prevalent among individuals aged 30–50 years, with a male‐to‐female ratio of approximately 2:1 [1]. LDH is a leading cause of LBP. The condition results from progressive degeneration of intervertebral disc (IVD) components, including the annulus fibrosus, nucleus pulposus, and connective tissue, and can be broadly defined as the bulging or protrusion of the nucleus pulposus material into the spinal canal [3]. Common symptoms of LDH, driven by nerve compression, include back pain, leg pain, numbness, diminished reflexes, and muscle weakness. In severe cases, it may progress to cauda equina syndrome [4]. The annual incidence of LDH is approximately 1%–3%, with the highest prevalence among individuals aged 30–50 years. Nevertheless, many patients with disc herniation remain asymptomatic. Imaging studies report detection rates of 30% in people in their twenties and up to 84% in those in their eighties [5].

Patients with LDH often experience severe pain that markedly diminishes their quality of life, alongside considerable anxiety about the condition itself [6]. This pain is typically progressive, prompting most affected individuals to pursue treatment [7]. Conservative management currently serves as the primary clinical approach for LDH. Key strategies involve modifying activities and implementing physical therapy. Surgery is reserved for cases where conservative methods prove ineffective or when severe neurological deficits emerge. Certain studies have also documented unfavorable surgical outcomes; for example, Carragee et al. [8] reported that only 43% of patients undergoing spinal fusion for lumbar intervertebral disc degeneration (IVDD) achieved a minimum acceptable outcome. Analgesics available without prescription, including acetaminophen and nonsteroidal anti‐inflammatory drugs (NSAIDs), are frequently advised [9]. Although celecoxib, an NSAID, can substantially alleviate pain following lumbar microdiscectomy [10], systematic evidence confirming its benefits for improving daily function remains scarce, and it poses a risk of gastrointestinal bleeding or perforation [11]. Furthermore, as a muscle relaxant, eperisone not only inhibits mono‐ and polysynaptic reflexes at the spinal and supraspinal levels but also regulates skeletal muscle blood supply [12].

Traditional Chinese medicine (TCM) is widely applied in the treatment of IVD herniation. Herbal preparations such as Duhuo Jisheng Decoction, used to dispel wind‐dampness and tonify the liver and kidneys [13], alongside individual herbs like *Eucommia ulmoides* (Du Zhong) and *Achyranthes bidentata* (Niu Xi), which strengthen tendons and bones, are commonly prescribed in clinical practice [14, 15]. In contrast, modern Chinese patent medicines are derived from natural ingredients through standardized manufacturing processes. Jintiange (JTG) capsule, a novel drug class, has been approved by the China Food and Drug Administration (CFDA) as a substitute for natural tiger bone [16]—a well‐known TCM historically employed for dispelling wind, alleviating pain, strengthening tendons and bones, treating bone atrophy, and managing skeletal disorders such as rheumatoid arthritis (RA) and osteoporosis (OP) [17]. JTG is widely used for OP; a recent meta‐analysis indicated that it effectively improves lumbar spine bone mineral density (BMD) in patients with primary OP [18]. Studies have demonstrated that JTG attenuates bone loss in rats, alleviates osteoarthritis (OA) and RA, and reverses alterations in serum metabolic profiles associated with collagen‐induced arthritis [19, 20]. Shen et al. reported that JTG prevented bone loss in ovariectomized (OVX) rats by regulating the BMP and Wnt/β‐catenin pathways and suppressed osteoclastogenesis through the modulation of osteogenic differentiation in bone marrow mesenchymal stem cells (BMSCs) and inhibition of the NF‐κB pathway [21]. Meanwhile, Pan’s research group found that warm acupuncture relieved pain and inhibited inflammatory responses in rats with noncompressive LDH induced by autologous nucleus pulposus transplantation, effects potentially linked to the suppression of the p38MAPK/NF‐KB pathway [22]. Meanwhile, emerging evidence directly supports the involvement of JTG capsule in this pathway: A recent study demonstrated that JTG inhibits JNK/p38/ERK phosphorylation and p65 nuclear translocation, thereby blocking MAPK/NF‐KB signaling pathway activation [23]. Additionally, JTG has been shown to function through the NF‐KB signaling pathway in the context of OA [20]. Based on the literature and the recognized role of inflammatory mediators as key drivers of pain in LDH, we hypothesize that JTG capsule may delay disc herniation by modulating inflammatory responses. However, the specific efficacy of JTG capsule in treating LDH remains unclear. To systematically evaluate the quality of available evidence, we adopted the Grading of Recommendations Assessment, Development and Evaluation (GRADE) system. The current evidence supporting these therapeutic benefits is of low quality, primarily classified as GRADE 1C or 2C.

This project addresses the pressing lack of standardized, safe treatments for LDH. Its primary goal is to alleviate pain, numbness, and related symptoms caused by LDH in clinical practice. To this end, we will conduct a high‐quality, single‐center, randomized, parallel‐group trial with a 1:1 allocation ratio under a superiority framework, aiming to clarify the clinical efficacy of JTG capsule for LDH and thereby offer patients an effective and safe treatment option.

## 2. Method

### 2.1. Study Design

This is a high‐quality, randomized, single‐center, positive‐controlled clinical trial designed to evaluate the efficacy and safety of JTG capsule in alleviating pain associated with LDH. Patients will be randomly assigned in a 1:1 ratio to the experimental group (JTG + celecoxib + eperisone) or the positive control group (celecoxib + eperisone), with no placebo administered to the control group. Both groups will receive standardized health education and undergo an 8‐week treatment period, followed by a 4‐week treatment‐free follow‐up. The final follow‐up assessment will be conducted at Week 12. Figure 1 provides a concise illustration of the participants’ flow through the study. As a protocol paper prepared in 2026 based on the 2023 registered protocol, this manuscript is reported in accordance with the Standard Protocol Items: Recommendations for Interventional Trials (SPIRIT) 2025 guidelines [24], and the checklist is available in Supporting File A. The adoption of SPIRIT 2025 for this manuscript reflects current reporting best practices and does not imply that the original 2023 registered protocol prospectively followed these guidelines.

**FIGURE 1:**
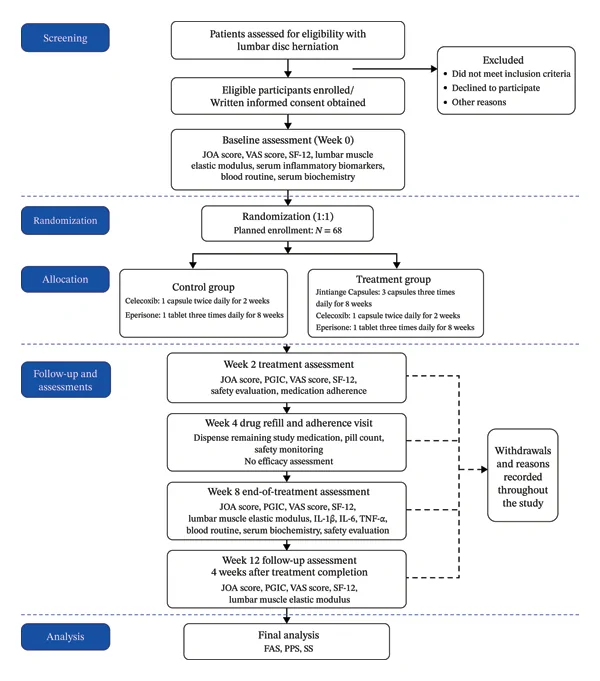
Flow diagram of the study design. Eligible participants will be randomly assigned in a 1:1 ratio to receive either Jintiange capsules plus celecoxib and eperisone or celecoxib and eperisone alone. The treatment period will last 8 weeks. Efficacy and safety assessments will be performed at baseline (Week 0), Week 2, and Week 8. A dedicated medication‐refill and adherence visit will be conducted at Week 4 without efficacy assessment. The final follow‐up assessment will be performed at Week 12, four weeks after treatment completion. JTG, Jintiange capsule; JOA, Japanese Orthopaedic Association; VAS, visual analog scale; FAS, full analysis set; PPS, per‐protocol set; SS, safety set.

### 2.2. Patient Recruitment

Participants will be recruited from the First Affiliated Hospital of Anhui University of Chinese Medicine. Recruitment will involve identifying potential candidates during follow‐up visits at spine specialty or discopathy clinics, displaying posters throughout the hospital, and launching social media campaigns. At each site, qualified study coordinators will perform an initial prescreening of interested individuals to determine preliminary eligibility. Attending physicians will then conduct a formal evaluation to confirm eligibility. Researchers will provide eligible participants with a comprehensive explanation of the trial procedures, including treatment protocols, and will address all questions and concerns. Written informed consent will be obtained to formally enroll participants. For individuals deemed ineligible or who decline participation, we will record basic demographic information and the reasons for nonparticipation. Patients will be assessed before treatment begins, and again at 2 and 8 weeks during the treatment period. A follow‐up assessment will occur 1 month after the 8‐week treatment course is completed. The specific assessments for each visit are detailed in Table 1.

**TABLE 1:** Schedule of enrollment, interventions, and assessments.

Time point	Admissions		Follow‐up			
	Screening	Baseline (Day:‐ 2 to 0; Week 0)	Week 2 (±2 days)	Week 4 (±2 days)^†^	Week 8 (±2 days)	Visit 3^∗^
Enrollment						
Admissions	√	—	—	—	—	—
Qualification screening	√	—	—	—	—	—
Informed consent	√	—	—	—	—	—
Intervention						
Jintiange capsules	—	√	—	√	—	—
Celecoxib capsules	—	√(2 weeks only)	—	—	—	—
Eperisone tablets dispensed	—	√	—	√	—	—
Drug compliance monitoring						
Returned medications/pill count	—	—	√	√	√	—
Therapeutic effect evaluation						
Japanese Orthopaedic Association Scores (primary outcome)	—	√	√	—	√	√
Visual Analog Scale (secondary outcome)	—	√	√	—	√	√
SF‐12 (secondary outcome)	—	√	√	—	√	√
PGIC (secondary outcome)	—	—	√	—	√	√
Elastic modulus of waist muscles (secondary outcome)	—	√	—	—	√	—
Laboratory and safety assessments						
IL‐1β, IL‐6, TNF‐α	—	√	—	√	√	—
Blood routine	—	√	—	√	√	—
Serum biochemistry	—	√	—	√	√	—
Adverse‐event and safety monitoring	—	√	√	√	√	√
Data recording						
Combined therapy	√	√	√	√	√	√
Electronic CRF	√	√	√	√	√	√

*Note:* Schedule of enrollment, interventions, assessments, and data collection across the study timeline. Symbols: “✔” indicates that the procedure/assessment is performed at that time point; “—” indicates that the assessment is not performed at that visit. PGIC is administered only at postbaseline efficacy visits (Weeks 2, 8, and 12), because it requires participants to rate their current overall condition relative to their pretreatment status.

^†^Week 4 is a dedicated drug refill visit for dispensing the remaining 4‐week supply of Jintiange capsules and eperisone, along with medication return, pill count, and safety monitoring; no efficacy outcomes are collected at this time point.

^∗^Week 12 is the final follow‐up visit conducted 4 weeks (±4 weeks) after the completion of the 8‐week treatment period (i.e., at Week 12 postrandomization). The ±4‐week window is permitted to enhance follow‐up completion rates in clinical practice. SF‐12, 12‐Item Short Form Health Survey (the short‐form version of SF‐36).

Abbreviations: CRF, case report form; IL, interleukin; IL‐1β, interleukin‐1β; IL‐6, interleukin‐6; JOA, Japanese Orthopaedic Association; TNF, tumor necrosis factor; TNF‐α, tumor necrosis factor‐alpha; VAS, Visual Analog Scale.

Laboratory sample collection note: Blood samples for routine safety laboratory tests (complete blood count [CBC], serum biochemistry including liver and kidney function, and coagulation function) and exploratory biomarker tests (IL‐1β, IL‐6, TNF‐α) are collected at baseline (Week 0), Week 4 (drug refill visit), and Week 8 (treatment completion) only, as indicated in the table. Detailed collection, processing, and analytical procedures are described in Section 2.8.4.

### 2.3. Inclusion and Exclusion Criteria

#### 2.3.1. Inclusion Criteria

Participants must meet the following criteria:

1. Meeting the diagnostic criteria for LDH (clinical symptoms: LBP and/or lower limb pain with or without numbness, muscle tension; imaging confirmation: lumbar MRI or CT showing disc herniation classified as protrusion, extrusion, or sequestration according to the Fardon classification criteria at the L1–S1 levels), confirmed by two radiologists independently reviewing the images;
2. Duration of LBP ≥ 3 months.
3. Baseline total Japanese Orthopaedic Association (JOA) score between 12 and 20 (out of 29).
4. Pain Visual Analog Scale (VAS) score between 3 and 7 (3 ≤ VAS ≤ 7).
5. Age 18–65 years.
6. Voluntary participation in the study and signed informed consent.

Furthermore, given that LDH itself is an inflammatory‐mediated disease, all eligible patients meeting the diagnostic criteria are considered to have an underlying inflammatory response; therefore, no additional screening for inflammation/infection status is required at enrollment.

#### 2.3.2. Washout Period for Concomitant Medications

To minimize the potential confounding effect of prior analgesic use on baseline pain assessments and treatment response, a prospective washout period will be implemented. Patients who have used NSAIDs, corticosteroids, or other analgesics within 7 days prior to screening will be required to discontinue these medications and undergo a 7‐day washout period before baseline assessment and randomization. This 7‐day duration is based on the pharmacokinetic properties of commonly used NSAIDs (approximately 5 half‐lives, sufficient for > 95% drug elimination) and is consistent with standard practice in analgesic clinical trials. During the washout period, patients will be permitted to use acetaminophen (paracetamol) as rescue medication for severe pain, not to exceed 2000 mg per day, with the dose and frequency recorded in the case report form (CRF). Patients who cannot safely discontinue concomitant analgesics for medical reasons will not be excluded but will have their medication use documented; these patients will be included in a prespecified subgroup analysis to assess whether concomitant medication use modifies the treatment effect. The washout period will be clearly explained to participants during the informed consent process, and compliance will be monitored through medication diaries and pill counts at the baseline visit.

#### 2.3.3. Exclusion Criteria

Participants will be excluded based on the following:

1. Comorbidities such as spondylolisthesis, severe nucleus pulposus herniation causing cauda equina compression, clear indications for surgery, or unsuitability for conservative therapy.
2. Presence of other conditions causing LBP (e.g., ankylosing spondylitis).
3. Patients with a duration of LBP of less than 3 months (< 3 months).
4. Known allergy to the study medications or a history of being allergy‐prone.
5. Severe cardiovascular, cerebrovascular, hepatic, or renal diseases.
6. Psychiatric disorders or dementia that would impede compliance with the clinical study protocol.
7. Pregnancy or lactation.
8. History of gastric ulcer or chronic gastritis.
9. Concurrent participation in another clinical trial.
10. Use of NSAIDs, corticosteroids, or other analgesics within 7 days prior to the screening visit, unless the patient agrees to undergo a 7‐day washout period before baseline assessment. Patients requiring ongoing analgesic therapy for other medical conditions (e.g., cardiovascular prophylaxis with low‐dose aspirin) may be enrolled after the documentation of the medication and will be included in a stratified analysis.

To ensure the external validity and generalizability of the findings, the inclusion and exclusion criteria were designed to reflect the real‐world clinical profile of patients with disc herniation. In routine practice, most patients are either treatment‐naïve or have only received conventional Western medication or surgical interventions, with minimal prior exposure to JTG capsules or celecoxib. Therefore, enrollment was not restricted by previous use of these specific treatments. To mitigate potential bias from concomitant medication use, a stratified analysis will be conducted based on whether other medications were used in the past month, where appropriate. This approach ensures the robustness and reliability of the study outcomes.

### 2.4. Criteria for Study Discontinuation

After randomization, any participant who voluntarily withdraws or meets the termination criteria will be considered a dropout. If a participant chooses to withdraw, the investigator should arrange a final visit to collect all available safety and efficacy data whenever feasible. The investigator must also contact the participant or their family to confirm the reason for withdrawal and retrieve any unused study medication. Reasons for withdrawal primarily include protocol violation due to poor compliance, loss to follow‐up, termination by the investigator, and intolerable adverse events. Any withdrawal due to an adverse event must be documented in detail in the CRF.

To protect participant rights and ensure trial quality while minimizing resource loss, investigators must accurately record all reasons for withdrawal. The study will be terminated under the following circumstances:

1. The investigator determines that continued participation is detrimental to the participant’s health.
2. The participant or their legal guardian requests withdrawal.
3. Occurrence of a serious allergic reaction related to the study medication.
4. Appearance of adverse symptoms, signs, or laboratory abnormalities deemed by the investigator to warrant study termination.
5. The participant becomes pregnant during the study period.

### 2.5. Intervention Protocol

Eligible patients will be randomly assigned to one of two groups:

1. Control Group:
 Celecoxib: One capsule twice daily for 2 weeks.
 Eperisone: One tablet three times daily for 8 weeks.
2. Treatment Group:
 JTG capsules: Three capsules three times daily for 8 weeks.
 Celecoxib: One capsule twice daily for 2 weeks (same as control).
 Eperisone: One tablet three times daily for 8 weeks (same as control).
 Study design: This trial adopts a positive‐control design (rather than a placebo‐controlled design) to compare the efficacy difference between JTG capsule combined with Western medicine and Western medicine alone.

JTG capsules are manufactured by Xi’an Jinhua Pharmaceutical Factory, Jinhua Enterprise (Group) Co., Ltd. (National Medicine Approval No. Z20030080).

Celecoxib capsules are provided by Shijiazhuang Yiling Pharmaceutical Co., Ltd. (National Medicine Approval No. H20203297).

Eperisone tablets are manufactured by Eisai (China) Pharmaceutical Co., Ltd. (National Medicine Approval No. H20041061).

All study medications must be stored securely in a dry place to maintain their stability. The clinical trial site will designate a responsible person to supervise the dispensing and management of the study drugs. Quality control assessments conducted by the manufacturers verified compliance with the standards specified in the Chinese Pharmacopoeia (2020 Edition) [25].

During the treatment period, patients are prohibited from using Western medicines, Chinese herbal medicines, proprietary Chinese medicines, or topical therapies that could duplicate the effects of the study drugs or target the same conditions. If other permitted medications become necessary, their names, reasons for use, dosage, and treatment duration must be meticulously recorded in the case observation forms. This detailed documentation is essential for preserving the study’s integrity and ensuring the accuracy of its outcomes.

#### 2.5.1. Drug Dispensing and Compliance Monitoring

All study medications will be stored, dispensed, and accounted for by authorized clinical pharmacists or research nurses at the GCP pharmacy of the study site, in accordance with the institutional standard operating procedures (SOPs) and Chinese GCP requirements.

 Dispensing schedule: Celecoxib (2‐week course) will be dispensed as a single 2‐week supply at the baseline visit (Week 0). JTG capsules and eperisone (8‐week course) will be dispensed in two batches: a 4‐week supply at baseline (Week 0) and the remaining 4‐week supply at a dedicated drug refill visit at Week 4 (which involves no efficacy data collection). All dispensing transactions will be documented in the Investigational Drug Dispensing/Return Log, signed by both the dispenser and the participant.
 Compliance monitoring: Adherence will be monitored primarily by pill count at each visit (Week 2, Week 4 Drug Refill Visit, and Week 8). Participants will be instructed to return all unused medications (including empty blisters/packages and any leftover tablets/capsules) at each scheduled visit. The study pharmacist will count the returned medication and calculate the adherence rate as
(1) adherence rate%=total dispensed−total returnedexpected total intake×100%.

An adherence rate ≥ 80% will be considered satisfactory. In addition, each participant will receive a Medication Diary Card to record daily dosing times and will be queried about their intake at each visit. For participants with poor adherence (< 80%), additional adherence counseling will be provided. All pill count results, diary cards, and inquiry records will be archived in the participant’s study file as source documentation.

#### 2.5.2. Washout Period Procedures

During the 7‐day washout period, patients will be contacted by study coordinators via telephone at Day 3 and Day 7 to confirm medication discontinuation, assess pain levels, and document any rescue medication use. Patients who experience intolerable pain during the washout period may use acetaminophen (paracetamol) as rescue medication, not exceeding 2000 mg per day, with all use recorded in the CRF. Patients who are unable to complete the washout period due to severe pain will be withdrawn from the trial and offered standard clinical care. At the baseline visit (Day 0), patients will return their medication diaries and any unused medications; adherence to the washout will be verified by pill count and diary review. Patients who have not adhered to the washout requirements (i.e., have used prohibited NSAIDs or corticosteroids during the washout period) will be rescreened after an additional 7‐day washout period, at the investigator’s discretion.

### 2.6. Randomization

This study adopts a positive‐control design without a placebo group. The randomization sequence was generated by an independent researcher using SPSS 28.0 software [3, 26, 27], stratified by herniation type (protrusion, extrusion, or sequestration), and arranged according to the chronological order of participant enrollment. The resulting randomization code table will be transferred to the statistician by a dedicated randomization coding officer and must be reviewed and approved by the sponsor.

After all data are entered into the electronic data capture system, they will be locked following data query and validation. Once the database is locked, the principal investigator and statistician will jointly determine the group assignments and interventions for each participant, based on which statistical analysis will be conducted. In the event of emergencies such as serious adverse events (SAEs), an emergency procedure may be initiated after confirmation and signing by the principal investigator. This procedure must be reported to the sponsor and the principal investigator within 24 h, with a written explanation of the reason.

### 2.7. Sample‐Size Determination

The sample‐size calculation was based on the change in the 29‐point JOA score, the primary efficacy endpoint of this study. Assuming a two‐sided significance level of 0.05 (Zα/2 = 1.96), 90% statistical power (Zβ = 1.28), equal allocation between the two groups, a common standard deviation (*σ*) of 2.84, and a prespecified clinically relevant between‐group target difference (*δ*) of 2.5 JOA points, the required number of evaluable participants was calculated using the two‐sample comparison‐of‐means formula:

(2) n=Zα+Zβ2∗2σ2δ2.

Substitution of these parameters yielded *n* = 27.09 evaluable participants per group. To account for an anticipated 20% dropout rate, the required enrollment was inflated as 27.09/(1 − 0.20) = 33.86 participants per group. Rounding upward, 34 participants will therefore be enrolled in each group, resulting in a total planned sample size of 68 participants.

The 2.5‐point value is used as the prespecified target difference for sample‐size planning rather than as an established minimum clinically important difference (MCID) for the 29‐point lumbar JOA score in LDH, because an LDH‐specific anchor‐based MCID for this exact instrument has not been firmly established [28]. To assess the sensitivity of the sample‐size requirement to this assumption, we additionally calculated the required enrollment across plausible target differences while retaining the same standard deviation, alpha, power, allocation ratio, and 20% dropout rate (Table 2).

**TABLE 2:** Sample‐size sensitivity analysis.

Target difference δ (JOA points)	Required evaluable n/group	Enrollment/group after 20% dropout	Total enrollment
2.00	42.33	53	106
2.25	33.45	42	84
**2.50**	**27.09**	**34**	**68**
2.75	22.39	28	56
3.00	18.82	24	48

*Note:* Sample‐size estimates were calculated using different assumed between‐group differences in the Japanese Orthopaedic Association (JOA) score. Calculations assumed a common standard deviation of 2.84, a two‐sided significance level of 0.05, 90% statistical power, a 1:1 allocation ratio, and an anticipated dropout rate of 20%. The bold values indicate the selected sample‐size scenario based on an assumed between‐group difference of 2.50 JOA points, corresponding to a total planned enrollment of 68 participants.

### 2.8. Data Collection Procedures

#### 2.8.1. Primary and Secondary Outcomes

To reduce measurement variability arising from the open‐label design, outcome assessors underwent standardized training in JOA administration before study initiation, following a unified operational manual. Standardized written instructions will also be used for participant‐completed VAS, SF‐12, and Patient Global Impression of Change (PGIC) assessments. PGIC will be completed directly by participants rather than rated by investigators. Because participants and clinical assessors are not blinded to treatment allocation, the risk of expectation and detection bias cannot be entirely eliminated.

##### 2.8.1.1. Blinding of Outcome Assessors

To further mitigate detection bias arising from the open‐label design, outcome assessors responsible for administering the JOA, VAS, and lumbar muscle elastic modulus measurements will be blinded to treatment allocation. These assessors will have no involvement in any other aspect of the trial, including treatment administration, patient management, or safety monitoring, and will be physically separated from the treatment team. Participants will be reminded before each assessment not to disclose their group assignment to the assessor. The success of assessor blinding will be evaluated at the end of the trial by asking assessors to guess each participant’s treatment allocation; the proportion of correct guesses will be compared to chance (50%) using a binomial test. This assessment will be reported in the final study publication to allow readers to evaluate the integrity of blinding. All assessors have received standardized training on scale administration following a unified operational manual, as previously described.

##### 2.8.1.2. Scale Language Versions and Validation

All scales used in this study have been administered in their validated simplified Chinese versions. It is important to distinguish between two distinct instruments developed by the Japanese Orthopaedic Association: (1) the original 29‐point JOA scoring system used as the primary outcome in this trial and (2) the Japanese Orthopaedic Association Back Pain Evaluation Questionnaire (JOABPEQ), a 25‐item, five‐domain questionnaire used for the SF‐12 validation in this study. The JOABPEQ was translated and culturally adapted into simplified Chinese following standard cross‐cultural adaptation guidelines. Psychometric testing demonstrated excellent internal consistency (Cronbach’s *α* = 0.886) and high test–retest reliability (ICC range: 0.951–0.977). The simplified Chinese JOABPEQ has been confirmed as a reliable and valid instrument for measuring multidimensional functional status in Chinese patients with LBP [29]. The VAS is a widely used pain assessment tool whose Chinese version has demonstrated good reliability and validity in Chinese populations. The SF‐12 Health Survey has also been validated in Chinese populations, with good internal consistency and test–retest reliability (range: 0.67–0.82); the standard SF‐12 explained 82% and 89% of the variance of the SF‐36 PCS and MCS scores, respectively [30, 31]. The PGIC is a single‐item patient‐reported measure of overall change relative to pretreatment status. A standardized Chinese version of the PGIC item will be finalized before participant enrollment using forward translation, back‐translation, and pilot cognitive review, and identical wording will be used at all postbaseline visits. Because PGIC assesses change from the start of treatment, it will be administered at Weeks 2, 8, and 12 and will not be collected at baseline.

The primary outcome of this study was the change in the original 29‐point JOA scoring system for LBP (maximum score 29; lower scores indicate greater dysfunction), a widely used instrument for evaluating treatment efficacy in LDH [28]. This scoring system provides a comprehensive assessment based on four domains:

1. Subjective Symptoms: Including LBP, leg pain, and/or tingling.
2. Clinical Signs: Including straight leg raising test (including sciatic nerve traction sign), sensory disturbance, and motor disturbance.
3. Activities of Daily Living (ADL): Assessing seven common daily activities: turning over while lying down, standing, washing face/body, leaning forward, sitting (for approximately 1 h), lifting/holding objects, and walking.
4. Bladder Function.
 A lower total score indicates more severe LBP and related neurological dysfunction. Furthermore, the JOA score is used not only to assess baseline status but also, and more commonly, to evaluate treatment outcomes (particularly surgical efficacy). The recovery rate is frequently used in clinical practice to quantify the degree of improvement, calculated as follows:
(3) improvement rate=post−treatment score−pretreatment scorefull score of 29−pretreatment score×100%.

An improvement rate ≥ 75% is classified as excellent, while a rate < 25% is considered poor.

##### 2.8.1.3. Secondary Outcomes

The following secondary outcomes will be assessed:

1. PGIC: The PGIC will be used to assess each participant’s perceived overall change in symptoms and functioning relative to pretreatment status. At Weeks 2, 8, and 12, participants will answer the standardized question, “Compared with your status before starting study treatment, how is your overall condition now?” using the following seven ordered response categories: 1, very much improved; 2, much improved; 3, minimally improved; 4, no change; 5, minimally worse; 6, much worse; and 7, very much worse [32]. PGIC will not be administered at baseline because the instrument requires a retrospective comparison with the pretreatment state. The seven‐category response will be treated as an ordinal outcome rather than a continuous score. In a supportive responder analysis, participants reporting “very much improved” or “much improved” will be classified as having achieved a clinically meaningful global improvement.
2. VAS Score: The VAS will assess LBP and lower limb pain before and after treatment. Scores range from 0, indicating no pain, to 10, which signifies the most severe pain; higher scores reflect greater pain intensity. The primary outcome is the change in the low back and lower limb pain VAS score from baseline to the 8‐week assessment.
3. SF‐12: The 12‐Item Short Form Health Survey (SF‐12) is a widely used quality‐of‐life assessment tool, consisting of two summary components: the Physical Component Summary (PCS) and the Mental Component Summary (MCS). The SF‐12 PCS and MCS scores are norm‐based scores with a mean of 50 and a standard deviation of 10 in the general population. These scores are computed through a standardized algorithm that involves recoding and normalizing each of the 12 item responses…. The SF‐12 is a generic quality‐of‐life instrument distinct from the JOA scoring system; it is included as a secondary outcome to assess the broader impact of treatment on patients’ physical and mental well‐being. In this study, we will use the validated Chinese (HK) SF‐12 scoring algorithm derived from Hong Kong Chinese population data [30]. Given that SF‐12 scoring requires proprietary licensed software or authorized algorithms, the specific scoring syntax will be detailed in a separate statistical analysis document. Detailed scale items can be found in Supporting Information B.
4. Lumbar Muscle Elastic Modulus: This is an objective physical measure of the “hardness” or “stiffness” of the lumbar muscles. This study will primarily measure the elastic modulus values of the multifidus and erector spinae muscles.

Multifidus Muscle: The shear wave elastic modulus and muscle belly thickness of the multifidus muscle will be assessed before treatment and after 8 weeks [33]. A high‐frequency probe on an ultrasonic elastography diagnostic device (LOGIQ E20, GE HealthCare, Nasdaq: GEHC) will be used for all measurements. A single sonographer will acquire images and measure the shear wave elastic modulus and thickness of the bilateral multifidus muscles at the L4 vertebral level for every patient. Each measurement will be repeated three times, with the average value recorded.

Erector Spinae Muscles: The absolute difference in hardness between the left and right lumbar erector spinae muscles will be assessed before treatment and again after 8 weeks of treatment. A soft tissue hardness meter (OE‐220, Ito Co., Ltd., Japan) will measure the hardness bilaterally [34]. Each site will undergo three measurements, from which the average value will be derived. The absolute hardness difference is defined as |left‐side hardness–right‐side hardness|.

#### 2.8.2. Safety Outcomes

Throughout this study, participants’ vital signs—heart rate, respiration, and blood pressure—will be monitored before and after each treatment. Any potential drug‐related adverse reactions, including nausea, vomiting, gastric discomfort, flushing, and night sweats, will be closely observed. Blood samples for routine safety laboratory tests (CBC, liver and kidney function, and coagulation function) will be collected at baseline (Week 0), Week 4 (drug refill visit), and Week 8 (treatment completion). Table 1 provides the detailed collection schedule. For standardized collection, processing, analytical methods, and statistical handling of laboratory data, please refer to Section 2.8.4. All adverse events occurring during the study will be documented in detail, with evaluations of their severity and potential relationship to JTG capsules.

An adverse event refers to any unfavorable medical occurrence in a patient following administration of the study drug, regardless of whether it is causally related to the treatment. All adverse events arising during the trial, including laboratory abnormalities, must be thoroughly documented and investigated. Each event’s nature, severity, and relationship to the study drug should be evaluated and precisely recorded in the CRFs. Causality assessment between adverse events and the investigational product will follow the Technical Guideline for the Evaluation of Causality of Adverse Events in Drug Clinical Trials [35], issued in 2024 by the Center for Drug Evaluation (CDE) of the National Medical Products Administration (NMPA). Should any injury or SAE related to the trial occur during this clinical study, the participant’s physician and the hospital will provide necessary medical care.

##### 2.8.2.1. Adverse Event Adjudication Committee (AEAC)

To enhance the objectivity and consistency of safety evaluations, an independent AEAC has been prespecified in the protocol. The AEAC will consist of three clinically experienced physicians who are not involved in the conduct of this trial and who will remain blinded to treatment allocation throughout the adjudication process. The AEAC will review all adverse events reported during the trial, including SAEs, adverse events leading to discontinuation, and any events with uncertain causality. For each event, the AEAC will independently assess the following:

1. severity (using CTCAE v5.0 criteria),
2. causality (following the CDE/NMPA Technical Guideline for the Evaluation of Causality of Adverse Events in Drug Clinical Trials, 2024),
3. expectedness (whether the event is consistent with the known safety profile of the study medications), and
4. whether the event is attributable to the study regimen, concomitant medications, drug–drug interactions, or the underlying disease.

The AEAC will convene at predefined time points: (a) an interim review after completion of enrollment to identify any early safety signals, and (b) a final review after trial completion to adjudicate all reported AEs. Any disagreements among committee members will be resolved by consensus; if consensus cannot be reached, the majority opinion will prevail. The adjudication results will be documented in the final clinical study report. The establishment of a blinded AEAC is particularly important in this open‐label, combination‐regimen trial, where independent, blinded safety assessment helps mitigate potential reporting and detection biases that could affect safety interpretation.

All safety analyses in this trial are designated as exploratory. While we will follow the CDE/NMPA Technical Guideline for causality assessment and will compare adverse‐event frequencies between groups, the combination regimen limits unequivocal attribution of events to JTG alone. Results will be interpreted with caution, and any safety signals will be considered hypothesis‐generating for future research.

#### 2.8.3. Exploratory Biomarker Outcomes

To preliminarily explore the potential anti‐inflammatory mechanism of JTG capsules in treating LDH, we will measure serum levels of inflammatory cytokines (IL‐1β, IL‐6, and TNF‐α) before and after treatment. The detailed collection schedule, sample processing procedures, ELISA assay protocols, and statistical handling of these exploratory biomarker data are presented in Section 2.8.4 Laboratory Indicators (Part B). These biomarker data are designated as exploratory outcomes only, and results will be interpreted with caution as hypothesis‐generating for future mechanistic studies.

#### 2.8.4. Laboratory Indicators

To systematically present all laboratory assessments in this trial, we categorize them into routine safety laboratory tests and exploratory biomarker tests. Table 1 provides a comprehensive overview of the collection schedule for each laboratory indicator.

##### 2.8.4.1. Routine Safety Laboratory Tests

The following safety parameters will be assessed at baseline (Week 0), Week 4 (drug refill visit), and Week 8 (treatment completion):

1. CBC: including white blood cell count, red blood cell count, hemoglobin, and platelet count
2. Liver function tests: alanine aminotransferase (ALT), aspartate aminotransferase (AST), alkaline phosphatase (ALP), and total bilirubin (TBIL)
3. Renal function tests: serum creatinine (Cr) and blood urea nitrogen (BUN)
4. Coagulation function: prothrombin time (PT) and activated partial thromboplastin time (APTT)

Sample collection and processing: Peripheral venous blood samples (approximately 8 mL) will be collected into vacuum tubes containing appropriate anticoagulants (EDTA‐K2 for CBC; sodium citrate for coagulation; plain tubes without anticoagulant for serum biochemistry). Serum and plasma will be separated by centrifugation at 3000 rpm for 10 min within 2 h of collection. All samples will be analyzed at the central clinical laboratory of the First Affiliated Hospital of Anhui University of Chinese Medicine, which is accredited and participates in external quality assessment programs.

Analysis methods: Routine safety tests will be performed using standard automated analyzers (CBC: Sysmex XN‐9000, Sysmex Corporation, Japan; biochemistry: Beckman Coulter AU5800, Beckman Coulter, USA; coagulation: Stago STA‐R Max, Diagnostica Stago, France). All results will be reported in standard clinical units with corresponding reference ranges. Abnormal laboratory values will be flagged and reviewed by the principal investigator for clinical significance.

##### 2.8.4.2. Exploratory Biomarker Tests

To preliminarily explore the potential anti‐inflammatory mechanism of JTG capsules, serum levels of inflammatory cytokines will be measured as exploratory outcomes. The following biomarkers will be assessed:

 Interleukin‐1β (IL‐1β)
 Interleukin‐6 (IL‐6)
 Tumor necrosis factor‐α (TNF‐α)

Sample collection and processing: Peripheral venous blood samples (approximately 5 mL) will be collected from each participant at baseline (Week 0), Week 4 (drug refill visit), and Week 8 (treatment completion). Blood will be drawn into vacuum tubes without anticoagulant, allowed to clot at room temperature for 30 min, and then centrifuged at 3000 rpm for 10 min. The separated serum will be aliquoted into 1.5 mL Eppendorf tubes and immediately stored at −80°C until batch analysis.

Assay procedures: All cytokine levels will be measured in batch at the central laboratory of the First Affiliated Hospital of Anhui University of Chinese Medicine using commercially available human enzyme‐linked immunosorbent assay (ELISA) kits (CUSABIO, Wuhan, China), strictly following the manufacturer’s instructions. The corresponding catalog numbers are as follows: IL‐1β (CSB‐E08053h), IL‐6 (CSB‐E04692h), and TNF‐α (CSB‐E04740h). Each sample will be assayed in duplicate, and the mean value will be used for analysis. The lower detection limits are as follows: IL‐1β, 0.5 pg/mL; IL‐6, 0.7 pg/mL; TNF‐α, 1.0 pg/mL. Intra‐assay and interassay coefficients of variation will be maintained below 10% and 15%, respectively.

##### 2.8.4.3. Statistical Handling of Laboratory Data

Safety laboratory tests: Changes in laboratory values from baseline to Week 8 will be summarized descriptively (mean ± SD, median, range) for each group. The proportion of participants with clinically significant abnormalities will be compared between groups using Fisher’s exact test or chi‐square test, as appropriate. All safety analyses are designated as exploratory.

Exploratory biomarkers: Changes in cytokine levels from baseline to Week 8 will be compared between groups using Wilcoxon rank‐sum tests (given the expected non‐normal distribution). Correlation analyses (Spearman’s rho) between changes in cytokine levels and changes in JOA/VAS scores will be conducted to generate hypotheses for future mechanistic studies. No adjustment for multiple comparisons will be made for these exploratory analyses, and results will be interpreted with caution.

Integration with safety monitoring: All laboratory results will be reviewed by the principal investigator within 48 h of receipt. Any grade ≥ 2 laboratory abnormality (according to Common Terminology Criteria for Adverse Events [CTCAE] v5.0 criteria) will prompt a clinical evaluation, and the participant will be followed until the abnormality resolves or stabilizes. Serious abnormalities will be reported as adverse events and managed according to the protocol‐specified procedures described in Section 2.8.2.

### 2.9. Data Management

An independent researcher will design the CRFs to align with the trial protocol, integrating logic checks as specified in the data validation plan. Trained and qualified researchers utilized Epidata to construct the database and entered data into the electronic data capture system. Investigators must promptly address all data queries, whether generated automatically by the system, identified during monitoring visits, or raised by data managers. Once data entry and source data verification are complete, investigators are required to provide an electronic signature. Any subsequent modifications to the data will necessitate a new signature. After receiving joint approval from key stakeholders—the principal investigator, sponsor, statistician, and data manager—the independent researcher will lock the database and transfer it to the statistician for analysis. Upon completion of the statistical analysis, the data manager will close the EDC system, thereby finalizing the data management process. A Data Monitoring Committee (DMC) will not be established for this study, a decision justified by the trial design that omits an interim analysis.

### 2.10. Quality Assurance and Quality Control

This trial will adhere strictly to the ethical principles of the Declaration of Helsinki, the International Council for Harmonisation (ICH) Good Clinical Practice (GCP) guidelines, and applicable Chinese regulatory requirements [36, 37]. To ensure trial quality, all research personnel will receive specialized training on the study protocol and SOPs during the initiation phase. Following the release of SOPs, the project team will organize mandatory training for all staff, covering their specific roles in research, monitoring, data management, and drug management, to ensure proficiency in the relevant procedures. The sponsor or responsible institution will perform rigorous periodic quality audits in accordance with the SOPs. Any identified issues must be resolved promptly; serious noncompliance may lead to the suspension of individual investigators or entire study sites to maintain overall trial standardization and data reliability.

### 2.11. Trial Status

Subject recruitment for this clinical trial had not commenced as of the time of manuscript submission (July 2026). The start of recruitment has been delayed due to a combination of factors: (1) prolonged institutional administrative reviews following initial ethics approval in 2022, including contract negotiations, trial insurance procurement, and internal resource allocation; (2) residual disruptions from the COVID‐19 pandemic, which imposed staffing constraints and reprioritization of research activities at our institution during the postpandemic period; and (3) extended coordination with the manufacturer for the procurement and quality verification of investigational products. Recruitment is now anticipated to begin in October 2026. Importantly, no substantive modifications have been made to the core trial design, including eligibility criteria, intervention protocol, outcome measures, or statistical analysis plan. The Version 1.0 protocol (dated March 20, 2023) remains unchanged, and no formal amendment has been submitted to the ethics committee or the registration platform.

### 2.12. Statistical Analysis

The full analysis set (FAS) and per‐protocol set (PPS) will serve as the analysis populations for baseline and efficacy assessments, whereas the safety set (SS) will be used for safety evaluations. Missing data for the primary efficacy outcome will be imputed primarily using the last observation carried forward (LOCF) method. A sensitivity analysis employing multiple imputation will also be performed to assess the robustness of the findings. We will determine the proportion of randomized participants who completed the study or withdrew prematurely and will document their reasons for withdrawal. Additionally, we will categorize and analyze the reasons for exclusion from the PPS. A detailed comparison of demographic and baseline characteristics will be conducted, presenting continuous variables as mean, standard deviation, and range, and categorical data as frequencies and proportions.

Drug exposure and intergroup differences will be assessed using *t* tests. Efficacy will be determined through analyses of primary and secondary endpoints. For the primary endpoint, the change in the JOA score from baseline to Week 8 (and to Week 12 as a secondary time point) will be compared between groups using the analysis of covariance (ANCOVA), with the baseline JOA score included as a covariate and treatment group as a fixed effect. Given the single‐center design of this trial, no center effect is included as a covariate or fixed effect in this model. For secondary outcomes, the changes in lumbar and lower limb pain VAS scores at Weeks 2, 8, and 12 will be analyzed using a mixed model for repeated measures (MMRM), including treatment group, time, and the group‐by‐time interaction as fixed effects, with baseline VAS scores as covariates. Given the single‐center design of this trial, no center effect is included as a covariate or fixed effect in any of these models. The PGIC will be analyzed as an ordinal categorical outcome and will not be treated as a continuous variable. At Weeks 2, 8, and 12, the distribution of the seven PGIC response categories will be summarized using frequencies and percentages and compared between treatment groups using the Mann–Whitney *U* test. As a supportive responder analysis, the proportion of participants reporting “very much improved” or “much improved” will be compared between groups using the chi‐square test or Fisher’s exact test, as appropriate. SF‐12 and lumbar muscle elastic modulus outcomes will be analyzed using appropriate parametric or nonparametric methods according to their distribution and prespecified analysis plan. All analyses will be performed on both the FAS and PPS. The model will include treatment group, time, and the group‐by‐time interaction as fixed effects, with baseline values as covariates. All statistical analyses will be performed using SPSS 28.0, with a *p* value ≤ 0.05 indicating statistical significance, as predefined in the statistical analysis plan. All statistical analyses will be performed using SPSS 28.0, with a *p* value of ≤ 0.05 indicating statistical significance, as predefined in the statistical analysis plan. Prespecified subgroup analyses will be conducted to evaluate the treatment effect across different herniation types (protrusion, extrusion, and sequestration), using interaction tests to assess whether the treatment effect differs by subtype. No interim analysis will be conducted in this study.

### 2.13. Dissemination of Results

No study data will be disclosed to any third party without the sponsor’s prior written approval. All data and analyses will remain blinded until the study results are formally published. The final conclusions will be disseminated through publication in relevant scientific journals.

### 2.14. Patient and Public Involvement

The study and content of the trial protocol were discussed with the patient representative group associated with our research unit. Corrections and clarifications were made according to the received feedback.

### 2.15. Protocol Version and Trial Status

The clinical trial protocol was initially developed on March 20, 2023 (Version 1.0). This manuscript is a protocol paper written based on that registered version. During the preparation and revision of this manuscript (2026), we incorporated the most recently published reporting guidelines (SPIRIT 2025) and regulatory technical guidance (CDE/NMPA 2024) to enhance the methodological transparency and reporting quality of this protocol paper. It is important to emphasize that these reference updates are strictly editorial and reflect our effort to align with current best practices in protocol reporting; they do not constitute any substantive modification to the core scientific content of the registered Version 1.0 protocol, including the study design, interventions, primary/secondary outcomes, or statistical analysis plan. Consequently, no formal amendment has been submitted to the ethics committee or the registration platform, as the registered trial design remains unchanged. The ∼3‐year gap between registration (March 2023) and the anticipated start of recruitment (October 2026) arose from administrative delays following ethics approval, postpandemic operational constraints at the study site, and extended supply‐chain coordination for the investigational products, as detailed in Section 2.11. Critically, these delays affect only the operational timeline and do not alter any scientific aspect of the registered protocol. No participant outcome data are presented, as recruitment had not commenced at the time of manuscript submission.

## 3. Discussion

LBP, identified by the World Health Organization as one of the most burdensome diseases, is the most common type of pain limiting daily activities [38]. This condition is closely associated with IVD degeneration [39]. Its clinical manifestations include disc herniation, discogenic back pain, and spinal stenosis. While most patients respond well to conservative treatments such as limited bed rest, exercise, and specific injections [40], current clinical approaches—whether pharmacological or surgical—often fail to provide definitive resolution and carry a risk of recurrence. TCM has been applied for over a thousand years in preventing and treating IDD, underscoring the value of further research to develop more effective therapies.

TCM has been practiced for thousands of years across China and other Asian nations, valued for its unique strengths in safety, efficacy, and low rates of adverse effects. Many patients explore complementary and alternative medicine, and herbal remedies in particular have been reported to effectively relieve LBP and lower limb pain [41, 42]. A prior case‐control study indicated that VDR gene polymorphisms might influence the development of degenerative disc disease in the Chinese population, suggesting their potential as biomarkers for early detection [43]. Furthermore, orally administered herbal formulations are among the complementary and alternative therapies used for LBP and lower limb pain. Certain studies report that such herbal preparations benefit patients with LDH [44], although high‐quality randomized controlled trials in this area are still limited.

A genome‐wide association study (GWAS) of 54,358 to 766,345 participants identified modifiable risk factors for IVDD such as smoking, alcohol consumption, standing height, and educational attainment [45]. Individuals with higher grades of lumbar disc degeneration tend to report lower quality‐of‐life scores than healthy controls [46], and patients with disc herniation commonly exhibit reduced VAS and ODI scores [47]. Seyedhoseinpoor et al. further observed that the multifidus muscle was smaller in LBP patients than in asymptomatic individuals, although the erector spinae muscle size did not differ significantly [48]. Accordingly, the quality‐of‐life questionnaires, VAS scores, and LBP rating scale scores will be recorded at baseline and upon study completion, while lumbar muscle elastic modulus will be assessed at baseline and Week 8 (treatment completion).

Inflammatory factors are closely linked to disc herniation. A serological analysis of 392 individuals revealed elevated serum IL‐6 levels in IDD patients compared to healthy controls, along with increased IL‐6 in degenerated IVDs [49]. Gruber et al. further demonstrated that stimulation with IL‐1β and TNF‐α increases IL‐17 expression in degenerated human discs and enhances IL‐17 production by disc cells [50]. Extensive research has clarified the connection between inflammation and disc herniation. To investigate the relationship between JTG capsules and these inflammatory cytokines, blood samples will be collected. Therefore, to explore how JTG capsule may alleviate LDH‐related pain, we will collect blood samples at baseline, Week 4, and Week 8 to measure changes in inflammatory cytokines, including interleukin‐1β (IL‐1β), interleukin‐6 (IL‐6), and TNF‐α. This analysis is designated as an exploratory biomarker outcome (see Section 2.8.3 for detailed procedures), aiming to generate hypotheses regarding the potential anti‐inflammatory mechanism of JTG capsule, rather than to serve as a screening criterion for patient enrollment. Given the exploratory nature, these findings will not be used to draw confirmation.

### 3.1. Safety Evaluation as an Exploratory Endpoint

It is important to clarify that the safety assessment in this trial is exploratory in nature, and the interpretation of safety data is constrained by the study design in several critical ways.

First, the experimental group receives a triple‐drug regimen (JTG capsules + celecoxib + eperisone), whereas the control group receives a dual‐drug regimen (celecoxib + eperisone). This asymmetry means that any adverse event observed in the experimental group could theoretically be attributed to JTG, celecoxib, eperisone, interactions among any of the three agents, or the underlying disease itself. Regulatory guidance from the CDE/NMPA explicitly recognizes that when assessing causality, investigators must evaluate whether the adverse event “can be explained by the subject’s concomitant disease, concomitant medications, or other causes” In the context of a triple‐drug regimen, the presence of two concomitant background medications (celecoxib and eperisone) makes it methodologically impossible to exclude them—or their interactions with JTG—as the cause of any given adverse event. Even if adverse event frequencies differ between the experimental and control groups, such differences could reflect drug–drug interactions between JTG and the background therapy rather than the independent toxicity of JTG. These interaction effects would not manifest in a JTG monotherapy setting and therefore cannot be generalized to infer JTG’s standalone safety profile.

Second, the open‐label design compounds the attribution problem. Unlike a double‐blind trial where adverse event reporting is less susceptible to bias, the lack of blinding in this study means that both participants and investigators are aware of group assignments. This awareness may influence the reporting, recording, and interpretation of adverse events—for example, participants in the experimental group may be more vigilant about potential side effects of the “additional” medication (JTG), while investigators may scrutinize the experimental group more closely for safety signals.

Third, while we will systematically collect and report all adverse events, perform routine safety laboratory tests (CBC, liver and kidney function, coagulation function) at baseline, Week 4, and Week 8 and assess causality according to the CDE/NMPA Technical Guideline for the Evaluation of Causality of Adverse Events in Drug Clinical Trials (2024), the combination design does not allow for definitive attribution of observed adverse events solely to JTG capsules. We will adopt the following analytical strategy to maximize the informativeness of safety data despite these constraints: (1) compare adverse‐event frequencies between groups using appropriate statistical tests; (2) perform individual causality assessments following the CDE/NMPA guideline, explicitly documenting the presence of concomitant medications as a confounding factor; (3) conduct sensitivity analyses excluding adverse events that are clearly attributable to known side effects of celecoxib or eperisone; and (4) report all safety findings with appropriate caveats regarding the exploratory nature of the analysis.

Any safety signals identified in this trial will be interpreted with caution and will serve to generate hypotheses for future investigations. A double‐blind, placebo‐controlled, JTG monotherapy design—without concomitant celecoxib or eperisone—would be required to draw firm conclusions regarding the independent safety profile of JTG. We will explicitly state this limitation in the final study publication and will avoid making definitive claims about JTG’s standalone safety based on this trial alone.

### 3.2. Limitations Regarding Open‐Label Design and Subjective Outcome Measures

A significant limitation of this trial is the open‐label design combined with substantial reliance on subjective patient‐reported or assessor‐rated efficacy outcomes. The JOA score, VAS, PGIC, and SF‐12 may be influenced by participant expectations or investigator awareness of treatment allocation. Although lumbar muscle elastic modulus provides an objective physical outcome, it cannot fully offset the potential expectation and detection bias affecting the subjective measures. These limitations will therefore be considered when interpreting the magnitude and consistency of the observed treatment effects.

The potential impact of such biases is not trivial. A landmark meta‐analysis by Hróbjartsson et including over 13,000 participants demonstrated that nonblinded trials of subjective outcomes yielded effect sizes that were, on average, approximately 30%–40% larger than those from blinded trials [51]. In the context of our study, the prespecified clinically meaningful difference in the JOA score between groups was set at *δ* = 2.5. If 30%–40% of the observed between‐group difference was attributable to bias, a statistically significant finding might not necessarily reflect a true clinically meaningful treatment effect. Therefore, even if our results show the superiority of the combination regimen, the magnitude of the effect should be interpreted with caution, and the findings should be considered hypothesis‐generating rather than confirmatory.

To mitigate these biases to the extent possible, we implemented several measures: (1) all assessors underwent standardized training on scale administration to minimize inter‐rater variability; (2) the statistician responsible for data analysis was blinded to group allocation; and (3) we included exploratory objective biomarkers (IL‐1β, IL‐6, TNF‐α) and an objective physical measure (lumbar muscle elastic modulus) to provide supporting, less bias‐prone evidence. Nevertheless, these measures cannot substitute for a double‐blind, placebo‐controlled design. Future definitive trials should adopt rigorous blinding and, where feasible, incorporate objective imaging or biochemical endpoints alongside patient‐reported outcomes.

This study was designed with methodological input from experienced researchers. The study site was rigorously selected, and data quality assurance was prioritized. Stringent quality control measures and close monitoring of data collection were implemented to ensure the accuracy and reliability of the results. This careful attention to detail reflects a firm commitment to upholding high standards of scientific inquiry.

**Nomenclature**
ADL: Activities of Daily Living
ANCOVA: Analysis of Covariance
BMD: Bone Mineral Density
CDE: Center for Drug Evaluation
CFDA: China Food and Drug Administration
PGIC: Patient Global Impression of Change
CRF: Case Report Form
DMC: Data Monitoring Committee
EDC: Electronic Data Capture
FAS: Full Analysis Set
GCP: Good Clinical Practice
GRADE: Grading of Recommendations, Assessment, Development, and Evaluations
GWAS: Genome‐Wide Association Study
ICH: International Council for Harmonisation
IDD: Intervertebral Disc Degeneration
IL: Interleukin
IRB: Institutional Review Board
IVD: Intervertebral Disc
IVDD: Intervertebral Disc Degeneration
JOA: Japanese Orthopaedic Association
LBP: Low Back Pain
LDH: Lumbar Disc Herniation
LOCF: Last Observation Carried Forward
MCS: Mental Component Summary
NMPA: National Medical Products Administration
NSAIDs: Nonsteroidal Anti‐Inflammatory Drugs
OP: Osteoporosis
PCS: Physical Component Summary
PPS: Per‐Protocol Set
RA: Rheumatoid Arthritis
SF‐12: 12‐Item Short Form Health Survey
SOPs: Standard Operating Procedures
SPIRIT: Standard Protocol Items: Recommendations for Interventional Trials
SPSS: Statistical Package for the Social Sciences
SS: Safety Set
TCM: Traditional Chinese Medicine
TNF: Tumor Necrosis Factor
VAS: Visual Analog Scale
ELISA: Enzyme‐Linked Immunosorbent Assay

## Author Contributions

Yinghao Zhu: conceptualization and writing–original draft. Hao Lv: formal analysis and writing–original draft. Yinghao Zhu and Hao Lv contributed equally. Yi Wang: writing–review and editing. Xunlu Yin: conceptualization and supervision. Kaiming Li: conceptualization and supervision. Hengtong He: writing–review and editing. Tianlin Wang: writing–review and editing. Long Liang: conceptualization, supervision, and writing–review and editing.

## Funding

The authors declare that this research and/or publication received financial support. This study was funded by the National Natural Science Foundation of China (Nos. 82505632 and 82205155), the Anhui Provincial Natural Science Foundation (No. 2308085QH292), the Key Project for Inheritance, Innovation and Development of Traditional Chinese Medicine of Anhui Red Cross Foundation (No. 2025ZYZD02), the Young Scientific and Technological Talent Training Program of China Academy of Chinese Medical Sciences (No. ZZ16‐YQ‐006), the Traditional Chinese Medicine Science and Technology Project of Zhejiang Province (Nos. 2024ZL716 and 2023ZR122), and the Integrated Platform for Clinical Scientific Research in Traditional Chinese Medicine at Xiyuan Hospital, China Academy of Chinese Medical Sciences (XYZX0405‐12).

## Ethics Statement

The study obtained initial ethical approval from the Institutional Review Board of the First Affiliated Hospital of Anhui University of Chinese Medicine (IRB Approval No. 2022AH‐49) in 2022. Given that ethics approvals typically have a validity period of one year from the date of issuance, and the start of recruitment has been delayed to October 2026, the original approval will have expired by the time recruitment commences. Therefore, prior to the initiation of participant recruitment, we will submit a study progress report to the IRB to formally apply for an extension of the ethics approval validity period. Recruitment will only begin after receiving written re‐approval or an extension letter from the IRB. Written informed consent will be obtained from all participants prior to the commencement of the study.

## Conflicts of Interest

The authors declare no conflicts of interest.

## General Statement

Note: This article presents the study protocol only; no results are reported. Findings will be published separately upon trial completion.

This study employed a randomized, single‐center, parallel‐controlled design and rigorously followed the SPIRIT 2025 guidelines, which enhanced its methodological soundness.

The primary outcome is assessed using the JOA score, with additional measures—including the VAS, SF‐12, PGIC, and lumbar muscle elastic modulus—providing a multidimensional evaluation of efficacy.

We investigated the clinical utility of an integrated TCM and Western medicine regimen by combining JTG capsule with Western medications (celecoxib and eperisone) for LDH.

The open‐label design is a major limitation and is further compounded by the substantial reliance on subjective patient‐reported or assessor‐rated outcomes (JOA score, VAS, PGIC, and SF‐12). Since all primary and secondary efficacy outcomes are based on patient‐reported or assessor‐rated scales, the lack of blinding for both participants and investigators may introduce expectation bias (participants aware of their group assignment may over‐report improvement) and detection bias (investigators aware of allocation may unconsciously influence scale administration or interpretation). While we standardized assessment procedures, trained all evaluators, and blinded the statistician, these measures cannot fully eliminate bias inherent to open‐label subjective assessments. Findings should therefore be interpreted with caution, and future studies should consider a double‐blind, placebo‐controlled design with objective outcome measures.

Conducted only at the First Affiliated Hospital of Anhui University of Chinese Medicine, this single‐center study draws on a restricted sample source, which may limit the generalizability and external validity of the results.

Safety evaluation limitation: The experimental group in this trial receives a triple‐drug regimen (JTG capsules + celecoxib + eperisone), while the control group receives a dual‐drug regimen (celecoxib + eperisone). This design fundamentally limits the attribution of adverse events to JTG capsules alone for several reasons. First, observed adverse events in the experimental group could theoretically arise from JTG, celecoxib, eperisone, drug–drug interactions among the three agents, or the underlying disease itself—a complexity explicitly recognized in regulatory guidance, which identifies concomitant medications as a key factor that must be considered when ruling out alternative explanations for an adverse event. Second, the open‐label nature of the design compounds this limitation, as the lack of blinding may influence adverse event reporting through both participant expectations and investigator awareness. Third, even if adverse‐event frequencies differ between groups, such differences could reflect drug‐drug interactions between JTG and the background therapy rather than the independent toxicity of JTG—effects that would not manifest in a monotherapy setting.

To address these constraints, we will treat safety as an exploratory endpoint and will adopt a multipronged analytical approach: (a) compare adverse‐event frequencies between groups using Fisher’s exact test or chi‐square test; (b) perform individual causality assessments for each adverse event following the CDE/NMPA Technical Guideline for the Evaluation of Causality of Adverse Events in Drug Clinical Trials (2024), explicitly documenting the presence of concomitant medications in the experimental group as a factor complicating causality determination; (c) conduct sensitivity analyses to explore the robustness of safety findings; and (d) clearly distinguish between adverse events that are “possibly,” “probably,” or “definitely” related to the study regimen versus those attributable to other causes.

Nevertheless, we acknowledge that no analytical approach can fully overcome the inherent design limitation that the combination and open‐label design preclude definitive independent safety conclusions for JTG capsules. Any safety signals identified in this trial should be considered hypothesis‐generating and will require confirmation in future double‐blind, placebo‐controlled, JTG monotherapy studies. We will explicitly report this limitation in the final study publication and will refrain from making definitive claims about JTG’s independent safety profile based on this trial alone.

## Supporting Information

Additional supporting information can be found online in the Supporting Information section.

## Supporting information


**Supporting Information** Supporting File A provides the SPIRIT 2025 checklist for reporting the clinical trial protocol. Supporting Material B contains additional study assessment materials, including the SF‐12 questionnaire items referenced in the Methods section. These materials are provided to enhance the transparency and reproducibility of the study protocol.

## Data Availability

De‐identified individual participant data (IPD) collected during this trial—including demographic information, baseline characteristics, JOA scores, VAS scores, SF‐12 quality‐of‐life scores, PGIC responses, lumbar muscle elastic modulus measurements, laboratory safety data (CBC, liver and kidney function, coagulation function), and exploratory biomarker data (IL‐1β, IL‐6, TNF‐α)—will be made available upon reasonable request to the corresponding author (Long Liang, leungloongtcm@163.com) following the publication of the primary study results. Data sharing will be subject to approval by the Institutional Review Board of the First Affiliated Hospital of Anhui University of Chinese Medicine and compliance with applicable Chinese data protection regulations. Access will be granted under a data use agreement that ensures participant confidentiality and prohibits the use of the data for purposes other than the approved research. The study protocol and statistical analysis plan will be made publicly available alongside the primary publication. No data will be shared prior to the completion of the trial and the publication of primary results.
