# Supplementary material for: Study Protocol: Efficacy of Jintiange Capsule in Lumbar Disc Herniation: A Single‐Center, Randomized Controlled Trial
Source: Pain Res Manag. 2026 Oct 29;2026:4091533. doi: 10.1155/prm/4091533 (PMC13720337; doi:10.1155/prm/4091533)
Supplement: Supplementary file 1 [file PRM-2026-4091533-s001.zip › Supplementary Material B.docx]

SF-12

| **Evaluation content** | **Options** | **Score** |
| --- | --- | --- |
| 1、Overall, what do you think of your health condition? | Excellent□ Very good□ good□ Pretty good□ poor□ |  |
| 2、The following items might be your activities on a certain day. Now, do these activities have any impact on your health condition? If so, to what extent? First of all, moderate activities such as moving tables, cleaning, playing bowling or golf. Do these have any restrictions on your health condition? | There are many restrictions.□ Limit it a little bit□  There are no restrictions at all□ |  |
| 3、Climb a few flights of stairs. Are there any restrictions on your health condition now? | There are many restrictions.□ Limit it a little bit□  There are no restrictions at all□ |  |
| 4、Over the past four weeks, due to health reasons, have you at least been able to achieve the work you want to do? | "Can't□ Can□ |  |
| 5、Have there been any restrictions on your work or daily activities in the past four weeks due to health reasons? | No□ Yes□ |  |
| 6、Over the past four weeks, due to some emotional issues such as feeling depressed or anxious, have you been able to achieve the work you want to do? | "Can't□ Can□ |  |
| 7、Over the past four weeks, have you been unable to work or engage in other activities as usual due to some emotional issues such as feeling depressed or anxious? | No□ Yes□ |  |
| 8、To what extent has the pain hindered your normal work (including both outdoor and household chores) over the past four weeks? | Not at all□ Slight□ Moderate□ A little bit□ Have a serious impact□ |  |
| **9、**These questions are about how you have felt about some things around you over the past four weeks. For each question, please give an answer that is close to your feeling. How long have you felt calm over the past four weeks? | The entire time□ Most of the time□ For a very long time□  Some time□ A little time□  There's no time at all□ |  |
| **10、**How long have you been energetic in the past four weeks? | The entire time□ Most of the time□ For a very long time□  Some time□ A little time□  There's no time at all□ |  |
| **11、**How long have you felt unwell in the past four weeks? | The entire time□ Most of the time□ For a very long time□  Some time□ A little time□  There's no time at all□ |  |
| 12、How long have you felt unwell in the past four weeks? | The entire time□ Most of the time□ For a very long time□  Some time□ A little time□  There's no time at all□ |  |
